# Bioinformatics-Based Activities in High School: Fostering Students’ Literacy, Interest, and Attitudes on Gene Regulation, Genomics, and Evolution

**DOI:** 10.3389/fmicb.2020.578099

**Published:** 2020-10-14

**Authors:** Ana Martins, Maria João Fonseca, Marina Lemos, Leonor Lencastre, Fernando Tavares

**Affiliations:** ^1^Departamento de Biologia, FCUP-Faculdade de Ciências, Universidade do Porto, Porto, Portugal; ^2^CIBIO-Centro de Investigação em Biodiversidade e Recursos Genéticos, InBIO-Laboratório Associado, Universidade do Porto, Vairão, Portugal; ^3^MHNC-UP-Museu de História Natural e da Ciência, Universidade do Porto, Porto, Portugal; ^4^FPCEUP-Faculdade de Psicologia e Ciências da Educação, Universidade do Porto, Porto, Portugal

**Keywords:** bioinformatics, comparative genomics, gene regulation, high school, genomic literacy

## Abstract

The key role of bioinformatics in explaining biological phenomena calls for the need to rethink didactic approaches at high school aligned with a new scientific reality. Despite several initiatives to introduce bioinformatics in the classroom, there is still a lack of knowledge on their impact on students’ learning gains, engagement, and motivation. In this study, we detail the effects of four bioinformatics laboratories tailored for high school biology classes named “Mining the Genome: Using Bioinformatics Tools in the Classroom to Support Student Discovery of Genes” on literacy, interest, and attitudes on 387 high school students. By exploring these laboratories, students get acquainted with bioinformatics and acknowledge that many bioinformatics tools can be intuitive for beginners. Furthermore, introducing comparative genomics in their learning practices contributed for a better understanding of curricular contents regarding the identification of genes, their regulation, and how to make evolutionary assumptions. Following the intervention, students were able to pinpoint bioinformatics tools required to identify genes in a genomics sequence, and most importantly, they were able to solve genomics-related misconceptions. Overall, students revealed a positive attitude regarding the integration of bioinformatics-based approaches in their learning practices, reinforcing their added value in educational approaches.

## Introduction

Bioinformatics, understood as the use of computational resources to categorize massive raw data and retrieve meaningful information from datasets, has gained a primordial utility in scientists’ daily routine (Sadek, 2004). This paradigm of biological research cannot be disregarded when seeking to promote a scientifically informed society. Indeed, it demands the improvement of curricular and educational resources at middle and high school educational levels based on initiatives validated by focused science education research.

Learning by accessing online bioinformatics resources in the classroom has already proven to have a beneficial impact on students’ ability to build up and mobilize scientific contents, namely, related to drug resistance, phylogenetic trees, or genetic expression (Amenkhienan and Smith, 2006; Taylor et al., 2014; Newman et al., 2016; Machluf et al., 2017). In addition, the introduction of bioinformatics at high school enhances the learning of new information through novel technologies and recruits resources used in research laboratories, serving as a stimulus to spark students’ future interest in scientific careers (Kovarik et al., 2013; Machluf et al., 2017).

Despite the various initiatives across Europe to support teachers and students to integrate bioinformatics-based approaches in their classes, these remain sporadic and are still not implemented consistently. Recent studies have called attention to the importance of a joint effort by all stakeholders (e.g., research institutions, governmental entities, teachers, trainers, and researchers) to deliver an action plan that can lead to bioinformatics dissemination in schools in a wider, more structured and cohesive manner (Koch and Fuellen, 2008; Campbell and Nehm, 2013; Attwood et al., 2017). Recent reports call for more educational assessments to strengthen the positive impact of bioinformatics-based activities on students’ scientific and digital literacy, providing a rationale to incorporate bioinformatics in the curriculum (Dudley and Butte, 2009; Campbell and Nehm, 2013; Machluf and Yarden, 2013; Magana et al., 2014; Marques et al., 2014; Machluf et al., 2017).

This study aims to address the educational impact on high school students of a set of activities developed to introduce basic bioinformatics analysis used to deconstruct a bacterial genomic sequence into its coding genes (Martins et al., 2018a), using purposely tailored evaluation instruments. The main research question driving this investigation was: *are there significant changes in high school students’ scientific and digital literacy, interest, and attitudes toward gene regulation, genomics, and evolution after performing bioinformatics-based activities?*

## Materials and Methods

### Participants

The sample studied included a group of 387 students and 11 teachers from five public and private schools in Porto and Lisboa, Portugal. Fourteen 11th grade biology and geology classes (students’ age: 16–17 years old) and five 12th grade biology classes (students’ age: 17–18 years old), comprising 167 male and 220 female students, were involved in this study. Students’ average age was 16.34 ± 0.67 years. The study included an experimental group (*n* = 292) with 123 male students and 169 female students (average age: 16.27 ± 0.68 years) from 14 classes and a control group (*n* = 95) including 44 male students and 51 female students (average age: 16.54 ± 0.62 years) from five classes.

Students participated in the project as part of their science classes, and taking into account all ethical requirements, the project was institutionally approved by each school’s Directive Board. Upon entering the project, the participants were invited to take part in the study and informed of its nature and aims, being assured that all the data collected were to be processed and analyzed anonymously. Students were given the chance to participate in the project without participating in this specific study.

### Didactic Instrument: Bioinformatics Laboratories

A set of bioinformatics-based activities previously proposed by Martins et al. (2018a) to identify genes from a bacterial genomic sequence and disclose their genomic context in different species was chosen as the didactic instrument. A tutorial video^1^ provides teachers and students with a detailed road map of the sequential bioinformatics resources needed to deconstruct a 2 kb genomic region of *Escherichia coli* and determine its occurrence across different bacteria taxa and hypothesize about its evolution. Participants were initially instructed to select a particular *E. coli* strain (*E. coli* str. K-12 substr. MG1655, Accession number: NC_000913.3) and a specific 2 kb genomic region, to ensure that all of them would be working with the same genomic sequence, allowing for a more efficient teacher supervision and facilitating subsequent analysis. In fact, the 2 kb sequence proposed includes the *lac* operon, which is the paradigm used to introduce gene expression and regulation at the high school. This provides a meaningful curricular framing for these activities and is aligned with students’ previous knowledge. Furthermore, it is important to emphasize that implementing bioinformatics exercises framed within the curriculum was the main concern of the participant teachers. Currently, the Portuguese biology curricula for the 11th and 12th grades include contents related with DNA and protein synthesis (for example, transcription, translation, and start and stop codons), as well as evolution (Mendes et al., 2003), and genetic expression (Mendes et al., 2004). These topics are also comprised in the Next Generation Science Standards (NGSS; National Research Council, 2013). While these curricular topics are frequently focused on eukaryotic models, bacterial genomes were chosen as an educational instrument for this study having in mind that bacteria stand for the most represented domain in genome databases, reflecting its high taxonomic diversity, and may be easily recruited by ingenious bioinformatics platforms with graphical and user-friendly interfaces using a Windows or Mac browser. In addition, bacterial genomes are frequently restricted to a single replicon (i.e., the chromosome), besides having a small-sized haploid genome that favors comparative genomics and contributes to strengthen students’ knowledge on bacteria, fostering their motivation and interest on microbiology-related topics, presently poorly explored in high school.

**Figure 1:**
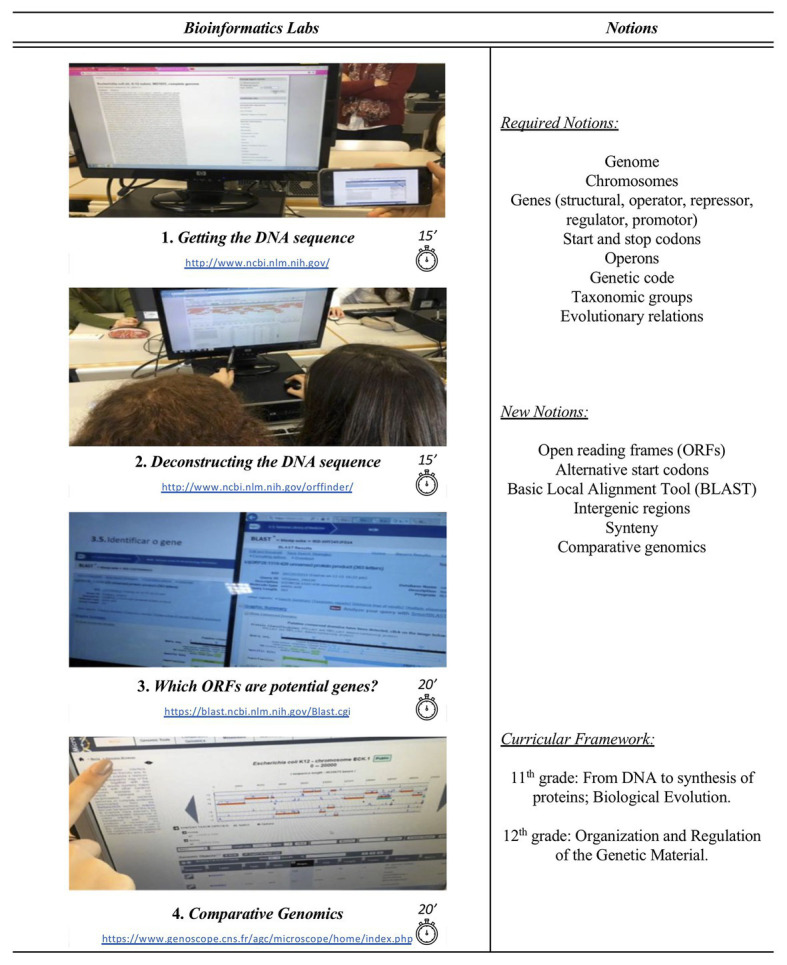
Bioinformatics laboratories framed within the curricular biology contents for high school to reinforce genomics topics currently required and to introduce new core concepts.

The bioinformatics resources used include the genome database, Open Reading Frames Finder (ORFfinder) and Basic Local Alignment Search Tool (BLAST) from the National Center for Biotechnology Information (NCBI; Altschul et al., 1990; Agarwala et al., 2018), and the genome browser of Magnifying Genomes (MaGe) that is part of MicroScope, a comparative genomics platform (Vallenet et al., 2013). Before starting with the *in silico* laboratories, the teachers work through basic and already known concepts, such as genome, genes, start codons, stop codons, and operons with the class, and introduce new notions, such as Open Reading Frames (ORFs), synteny, and comparative genomics (Figure 1; Martins and Tavares, 2018). This is particularly important since these new notions, presently absent from the curricula, are instrumental to understand the data retrieved by the students when performing the bioinformatics exercises proposed (Martins and Tavares, 2018).

### Research Design and Methodology

**Figure 2:**
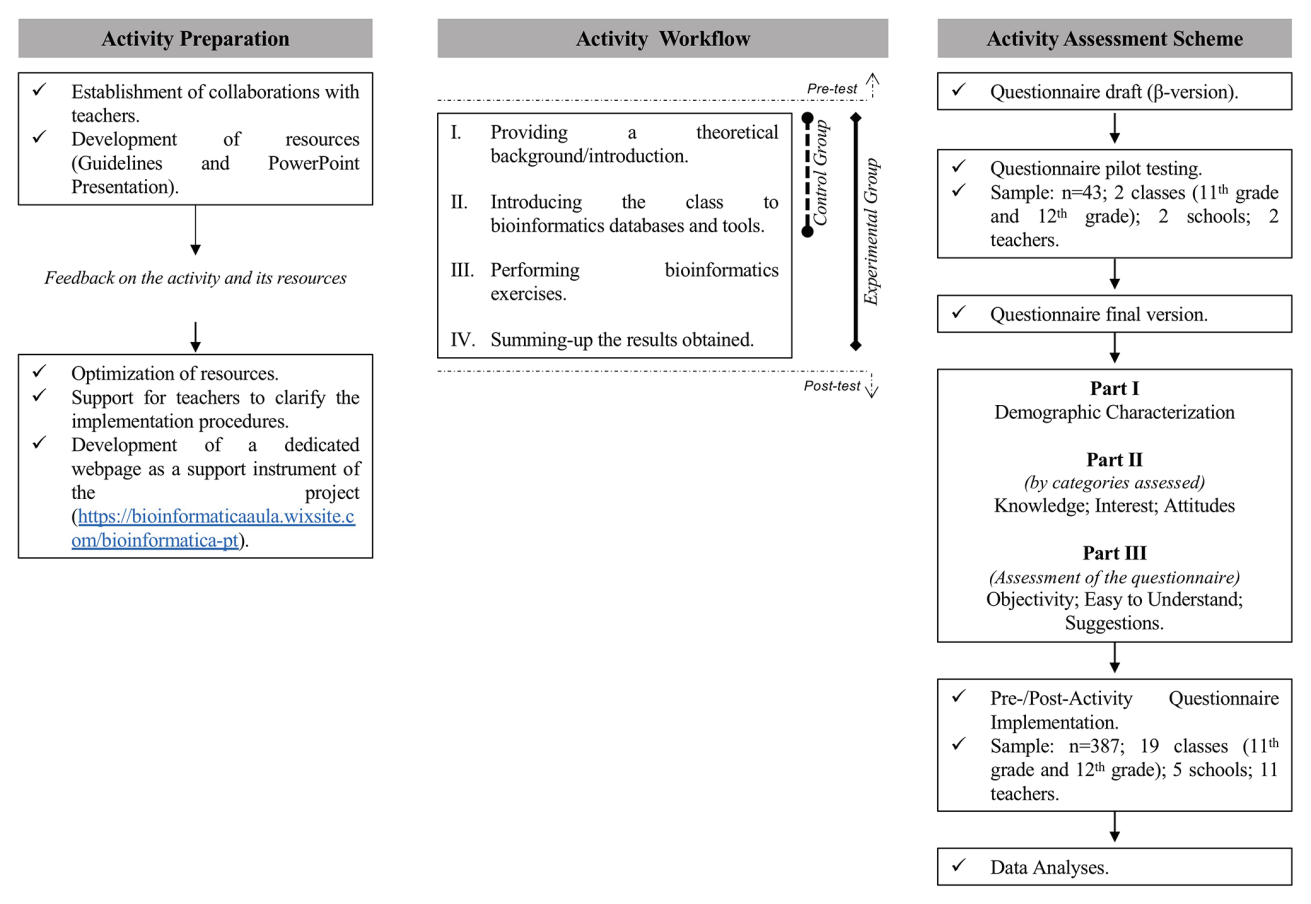
Experimental design for preparation, implementation, and assessment of bioinformatics-based activities.

To implement bioinformatics-based activities as a successful didactic instrument, it is crucial to engage both teachers and students in the selection of the activities to ensure that these are meaningful and adjusted to the curricular contents (Marques et al., 2014). In this regard, the design of the bioinformatics-based activities proposed by Martins et al. (2018a) took into account teachers’ contribution in revising and piloting the proposed educational resources with their students (Figure 2). To lighten the burden for teachers, a dedicated webpage^2^ was developed to provide them with resources that introduced the bioinformatics tools and the new concepts to be addressed.

The workflow of the bioinformatics activities includes four parts (Figure 2). Firstly, teachers provide the knowledge background about gene regulation, genomics, and evolution. Secondly, students are introduced to the bioinformatics databases and tools to be used, namely, NCBI database, NCBI ORFfinder, NCBI Blast, and MicroScope (MaGe). And thirdly, the bioinformatics exercises are performed. These exercises were set to meet the curricular requirements for the topic, and given the novelty of bioinformatics for these students (and teachers), guidelines were prepared to provide a comprehensible workflow to address the research questions outlined. This allowed to prevent students from becoming overwhelmed by the wide plethora of choices of links and commands available in the platforms mentioned before. In the fourth and final stage of implementation, the results obtained in each exercise were discussed with the students, and conclusions were drawn.

During the implementation of the activities, a member of the research team (Martins) was present to identify misconceptions and reasoning difficulties, as well as to check the participants’ engagement and interaction, and to carry out qualitative observations useful to improve the robustness of the interpretations made.

A quasi-experimental pre-/post-design, with a control and an experimental group, was set up. The control group included classes exclusively exposed to the first two parts of the intervention, i.e., the introductory lectures about the scientific questions and the bioinformatics databases and resources (Figure 2 – workflow I and II). In turn, the experimental group was exposed to the full set of the bioinformatics activities, i.e., from the introductory lectures to the bioinformatics laboratories and the interpretation of the results (Figure 2 – workflow I–IV). To mitigate possible bias effects, the control group classes were from the same schools, from the same education levels, and taught by the same teachers as the experimental group classes. The comparison between the performance of students in the control group and the experimental group was intended to test the educational impact of the practical bioinformatics-based activities. In this regard, the control group was taught only through expositive teaching (Figure 2 – workflow I and II), and the experimental group was exposed to the same lectures as the control group plus the practical component (Figure 2 – workflow I–IV).

### The Questionnaire

**Figure 3:**
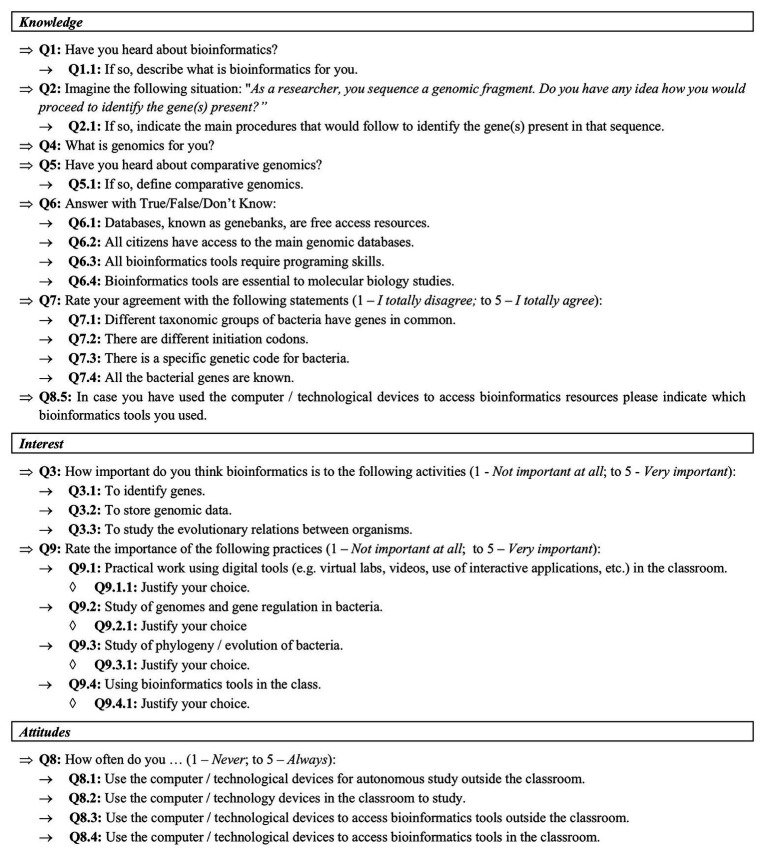
The questionnaire used in this study included demographic characterization of the participants and items to assess students’ knowledge, interest, and attitudes.

To assess the educational impact of integrating the mentioned bioinformatics-based activities in high school, a specific and comprehensive questionnaire including open-ended questions, dichotomous questions, and Likert-type scales was designed (Figure 3).

The questionnaire was structured according to three main dimensions: knowledge, interest, and attitudes. The knowledge-related questions (Q1, Q2, Q4, Q5, Q6, Q7, and Q8.5) aimed to characterize students’ literacy regarding gene regulation, comparative genomics, bioinformatics, and its usefulness for scientific research. Students’ interest (Q3 and Q9) was measured by their perception of the role of bioinformatics in tackling different biology research questions and by their awareness about the scientific disciplines addressed in the *in silico* activities, namely, genetics, genomics, and evolution. Students presently attending high school are part of the so called *iGeneration* (iGen), which is characterized by being highly motivated to use technology in their daily lives (Rosen et al., 2010; Quinn and Oldmeadow, 2013). Having this in mind, a question (Q8) was added to depict students’ attitudes toward the use of computer/technological devices to study and to assess their motivation to access bioinformatics tools inside or outside the classroom.

The questionnaire developed was piloted in two high school classes (*n* = 43 students; Figure 2), which, as recommended by several authors (Treece and Treece, 1982; Connelly, 2008; Johanson and Brooks, 2010), represent slightly over 10% of the universe of students included in the main research study. This procedure allowed to ensure that the students’ responses were not biased by a lack of comprehension of the questionnaire and also to prevent difficulties in deconstructing the answers to open-ended questions during the content analysis. Furthermore, it is important to highlight that in the final version of the measurement instrument, students were invited to rate the questionnaire regarding its objectivity and intelligibility, to guarantee that the questions were clear and well understood by all respondents.

Lastly, students from both the control group and the experimental group rated the questionnaire as being objective and easy to understand, which further emphasizes the adequacy of the validated version of the questionnaire.

### Data Analyses

Methods of descriptive and inferential statistics were used to analyze the pre-/post-test data. All statistical analyses were carried out using IBM’s Statistical Package for the Social Sciences (SPSS) version 24.

Independent samples *t*-tests and paired samples *t*-tests for a 95% confidence interval were used for five-point Likert-type scale data, and the effect size of mean differences registered with *t*-test was measured using Cohen’s *d* (Cohen, 1988). Data gathered through open-ended questions and dichotomous variables were analyzed using Chi-square and the McNemar tests, respectively, and considering the phi coefficient as the effect size measure (Pallant, 2007). Furthermore, to obtain a broader, more inclusive depiction of the effectiveness of the activities, while strengthening the interpretation of the outcomes of the analyses performed (Punch, 2009), it was decided to combine quantitative and qualitative methods of analysis, as has been suggested in similar studies (Gelbart et al., 2009; Fonseca et al., 2012; Machluf and Yarden, 2013). This methodology would avoid missing detailed information that cannot be retrieved exclusively from quantitative data (Johnson and Christensen, 2012).

In what concerns the qualitative data, a thematic content analysis of the participants’ responses to open-ended questions was performed with the purpose of producing a systematic description of the meaning of specific information gathered through the definition of coding categories (Schreier, 2012). This allowed to organize extensive answers to open-ended questions into fewer and more focused content categories (Weber, 1990; Krippendorff, 2004; Hsieh and Shannon, 2005). The analysis of the answers to the open-ended questions was performed according to the framework previously created by the authors in which specific categories of answers have been defined (Supplementary Figure 1). Regarding the open-ended question Q9, aimed to assess students’ interest, the subjective task value of Eccles and Wigfield (2002), Eccles (2005) that characterizes an expectancy–value model of achievement motivation was used as the theoretical framework underlying data analysis. Task value is related with the quality of the task, which influences the probability of it being select by an individual. In this study, the intrinsic/interest value (i.e., expected enjoyment of engaging in the task), the utility value (i.e., possible rewards from the task), and the cost of engaging in the activities were the dimensions considered when analyzing the students’ answers.

## Results and Discussion

### Students’ Literacy on Bioinformatics and Its Applications

It is consensual that an updated and edifying high school level education requires an attentive revision of the curricula aligned with the challenges of NGSS and capable to meet Science, Technology, Engineering and Mathematics (STEM) education (Wefer and Sheppard, 2008; Kovarik et al., 2013; National Research Council, 2013; Champagne Queloz et al., 2017). In this regard, bioinformatics is in a privileged position, due to the transdisciplinary approach it entails, by seeking a level of integration of different disciplines, such as biology, computer science, and mathematics, beyond the mere interdisciplinary relationship between them. It is therefore reasonable to acknowledge the importance of integrating bioinformatics in high school, as emphasized in several studies (Dudley and Butte, 2009; Machluf and Yarden, 2013; Magana et al., 2014; Marques et al., 2014; Machluf et al., 2017), even though there is scarce research on how to do it (Campbell and Nehm, 2013; Magana et al., 2014; Machluf et al., 2017). To measure the impact of educational initiatives using bioinformatics resources on high school students and to emend misconceptions and tailor adequate bioinformatics activities for successful learning, it is important to diagnose the knowledge students perceive to have about bioinformatics- and genomics-related concepts (Gelbart and Yarden, 2006; Gelbart et al., 2009; Form and Lewitter, 2011; Champagne Queloz et al., 2017).

**Figure 4:**
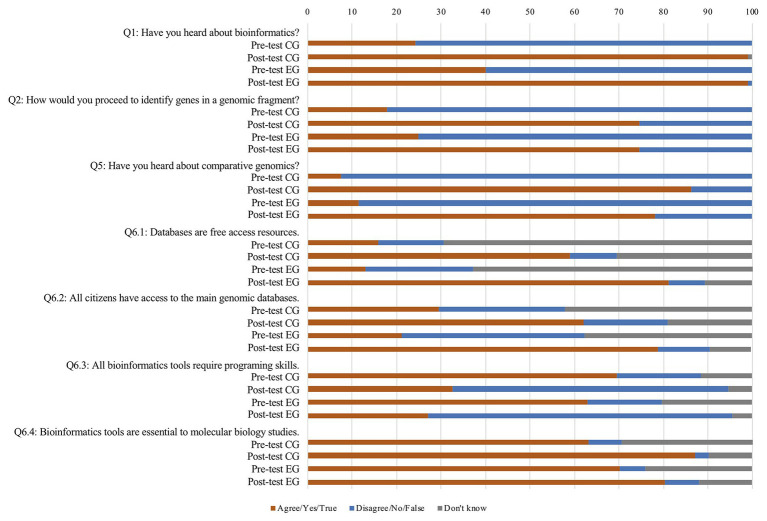
Students’ knowledge toward bioinformatics, gene regulation, and genomics.

In the universe of 387 high school students enquired in the present study, only a modest percentage (40.1% of the experimental group, 24.2% of the control group) revealed to have heard about bioinformatics in the pre-test (Q1), and most of the ones who did so could not define bioinformatics, admitting that their answer reflected the etymological meaning of the word. Following an expositive teaching session on bioinformatics and associated resources, such as databases and applications, in the post-test, the percentage of the students who revealed to have heard about bioinformatics raised consistently for both the experimental group (99.0%) and the control group (99.0%; Figure 4). Regardless of the fact that in the post-test most of these students linked bioinformatics to the etymology of the word: bio + informatics (60.9% of the experimental group, 73.6% of the control group), which undermines a truly sensible diagnostic of their understanding of bioinformatics, some students did mention specific aspects, such as data analysis, storage, and comparative genomics. The difference observed in this regard between the experimental and control groups (31.0% of the experimental group, 22.0% of the control group) may be explained by the fact that students in the experimental group carried out a set of bioinformatics exercises using the mentioned resources and used bioinformatics platform for comparative genomics, contrarily to their counterparts in the control group. This is particularly evident regarding comparative genomics, a completely new notion for the majority of the students, which was mentioned by 6.6% of the experimental group students and only by 1.1% of the control group students. Furthermore, students from both groups recognized that genebanks are open-access resources (Q6.1; 81.2% of the experimental group, 59.0% of the control group) and generally accessible to all citizens (Q6.2; 78.8% of the experimental group, 62.1% of the control group), suggesting an enhanced perception of what comprises a bioinformatics scientific toolbox and of their empowerment to access it (Figure 4). These findings, observed in other studies (Kovarik et al., 2013; Machluf et al., 2017), report for a motivational trigger of scientific literacy and STEM education. The higher percentage scores obtained with the experimental group indicate that complementing expositive teaching with hands-on *in silico* laboratories favors the acquisition of structural knowledge. This was a particularly relevant outcome that allows to dismiss the common misconception that bioinformatics analysis always requires programing skills. In fact, while initially, i.e., before the intervention, students from both groups (62.9% of the experimental group, 69.5% of the control group) agreed that programing skills would be needed to use bioinformatics tools (Q6.3; Figure 4), after the intervention, only 27.1% of the students from the experimental group and 32.6% of the control group agreed with this statement (Figure 4). These data indicate that while initially students associated bioinformatics analysis to a set of complex computer codes, after they were challenged with bioinformatics activities, they were able to acknowledge the panoply of bioinformatics applications with user-friendly interfaces tailored for web browsers that do not require programming competencies as has been highlighted by Martins et al. (2018b). Students were shown to be aware that bioinformatics tools are essential to molecular biology studies (Q6.4), in both the pre- and post-test (Figure 4). Still, in the post-test, there was a slight increment in the percentage of students who agree with this statement, suggesting that they confirmed their previous idea about the role of bioinformatics in molecular biology.

**Figure 5:**
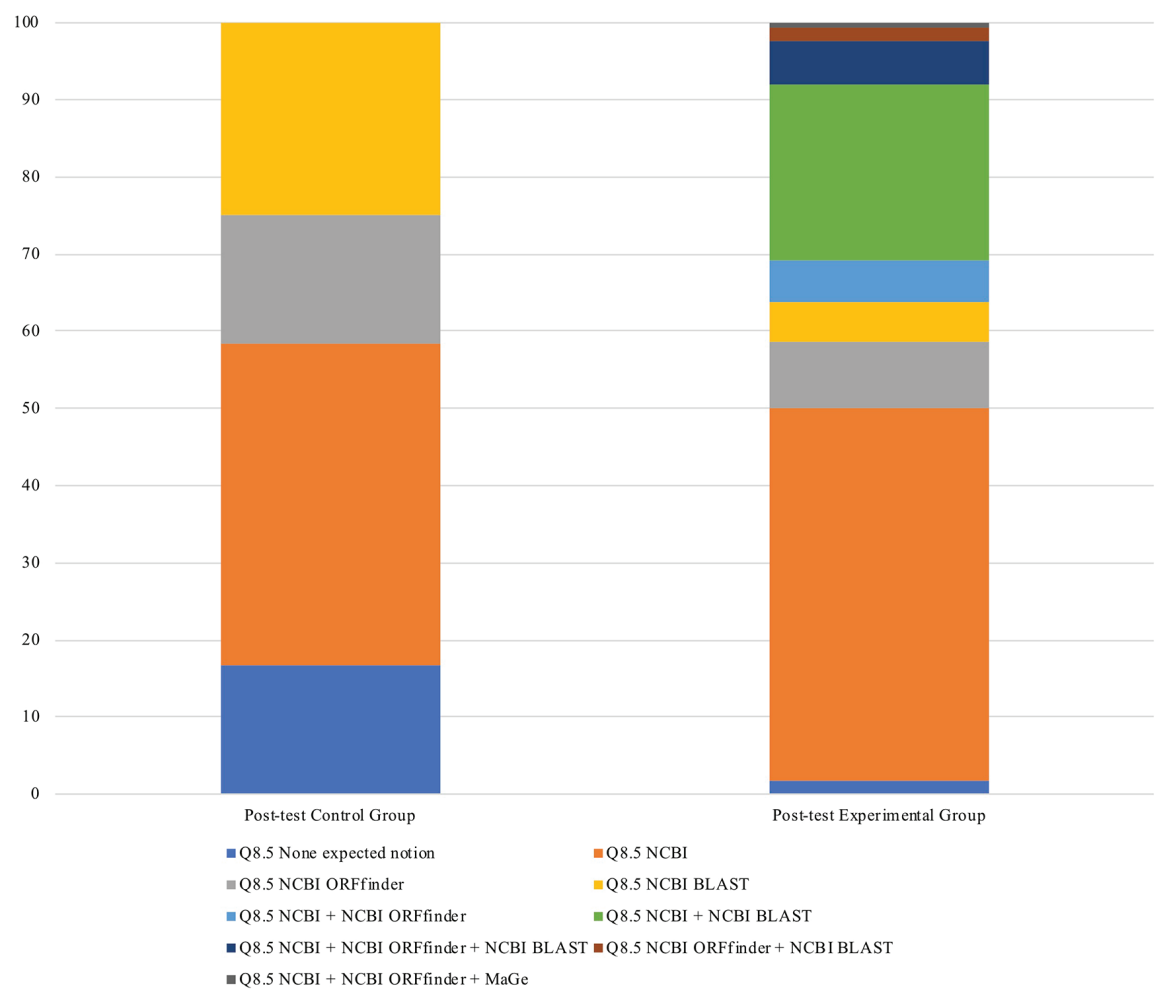
Bioinformatics tools mentioned by students to unveil genes from bacterial genomics sequences.

Following the intervention (i.e., post-test), when the participants were asked to “Indicate which bioinformatics platforms [they] used” (Q8.5), 16.7% of the students in the control group failed to mention any of the expected resources used during the intervention, namely, NCBI, NCBI ORFfinder, NCBI BLAST, and MaGe. This percentage dropped to 1.7% in the experimental group (Figure 5), indicating the positive impact of bioinformatics laboratories on students’ knowledge.

The bioinformatics exercises used in this study aimed to train the students on key procedures to identify genes from a genome sequence, as proposed by Martins et al. (2018a). Since the bioinformatics exercises were supported by a tutorial video comprising detailed guidelines and instructions,^3^ it was important to determine if the students’ performance actually contributed to enhance their knowledge on basics genome mining and did not resume to a mere mechanical procedure of following a recipe step by step. To address this question, the students were asked to describe the procedures that can be used to identify putative genes within a genomic DNA sequence (Q2, Q2.1). While in the pre-test, only a minority of the students in both groups (24.9% of the experimental group, 17.9% of the control group) claimed to know the procedures to deconstruct a DNA sequence into putative coding sequences, in the post-test, this percentage increased significantly (74.6% of the experimental group, 74.7% of the control group; Figure 4). As expected, the change between pre- and post-test is statistically significant for both groups (Supplementary Table 1). To fully elucidate if the students’ perceptions were aligned with their knowledge, a content analysis was carried out.

In this regard, a framework with three expected bioinformatics-related notions was defined: (1) “Getting the target DNA sequence in a database,” (2) “Looking for Open Reading Frames,” and (3) “Deciding which of the retrieved ORFs are likely to be genes running a BLAST.”

The pre-test content analysis regarding the answers to Q2.1 showed that students who admitted knowing how to identify putative genes from a genomic DNA sequence failed to mention any of the three notions. Instead, they mentioned, for instance, that “To unveil a DNA sequence we can perform an electrophoresis to determine the genes, looking at the gel bands in comparison to a known gene. Restriction enzymes may be needed in this procedure,” which was the most frequently recorded notion in the experimental group, and that it is possible to “Use the genetic code to identify the codons in a DNA sequence,” which was the most frequently recorded notion in the control group.

The post-test content analysis for the answers to Q2.1 revealed that 47.7% of the students in the control group did not mention any of the expected answers, 9.0% mentioned one of the expected answers, 41.8% mentioned two expected notions, and 1.5% mentioned all three expected notions. This trend improved in the experimental group, for which the percentage of students who mentioned one expected notion (14.3%) and all the expected notions (11.1%) was higher. Furthermore, the percentage of students who did not mention expected notions was lower in the experimental group than in the control group (38.6%).

Contrary to what was observed in the pre-test, in the post-test, students from both groups mentioned bioinformatics approaches, rather than wet laboratory techniques currently mentioned in their biology classes, such as electrophoresis and restriction enzymes. This outcome highlights the notion that, following a bioinformatics laboratory, most of the experimental designs envisioned by students to address a research question are based on a bioinformatics approach, instead of involving wet laboratory techniques that were already known to them. More than suggesting an enrichment of students’ scientific toolbox and the development of thinking skills, the intervention seems to narrow the gap between students’ school reality and what are common research practices nowadays, which is consistent with the educational benefits of bioinformatics reported in the literature (Gelbart and Yarden, 2006; Flanagan, 2013; Wood and Gebhardt, 2013). The data further suggest that when students are guided in the use of a wide variety of resources, they show to be capable to explore ideas and to interpret results in order to answer questions raised by the teacher (Kuhlthau et al., 2007).

### Students’ Knowledge on Gene Regulation and Genomics

**Figure 6:**
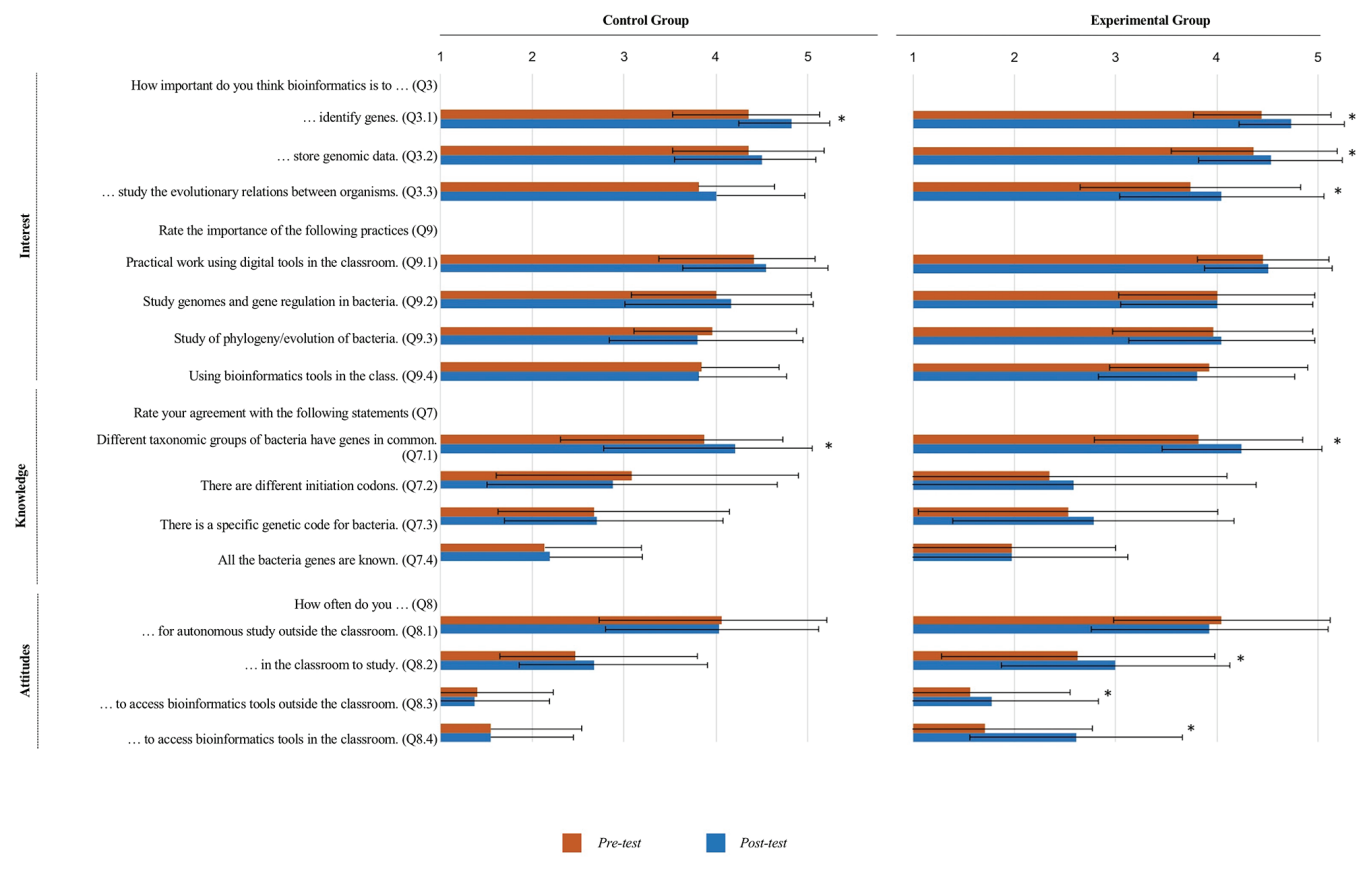
Students’ knowledge, interest, and attitudes toward the integration of bioinformatics in science curricula.

According to the educational theories proposed by Ausubel (1968) and Vygotskiĭ and Cole (1978), students’ prior knowledge, and in particular students’ misconceptions, is of crucial importance when learning a new issue. Several diagnostic instruments are available, in published research studies, that can be used to obtain guidelines for specific interventions to address these misconceptions (Klymkowsky et al., 2010; Tsui and Treagust, 2010; Gurel et al., 2015). Examples of students’ key misconceptions regarding basic genetic and genomics notions are already described in the literature and include the use of gene and genome as synonyms, the misunderstanding of the relationship between a gene and DNA, a misinterpretation of the association between a gene and gene regulation, and the idea that some organisms, such as bacteria and fungi, often do not have DNA (Lewis and Kattmann, 2004; Mills Shaw et al., 2008). Adding the relevance of addressing these misconceptions, the Portuguese biology curriculum for the 11th grade (Mendes et al., 2003) recommends the discussion of the concept of “codogene” – part of a gene, i.e., a triplet of DNA, which is contributing to mislead students on the definition of gene. Having in mind the reported misconceptions, the activities implemented in this study aimed to tackle notions related with genes, genomes, alternative start codons, and the genetic code. Participants of both groups agreed that different bacteria groups have genes in common (Q7.1) and were shown to be aware that not all bacteria genes are identified and characterized, and that genomics information is still missing for many species (Q7.4; Figure 6). Conversely, misconceptions related with gene structure and the features of the genetic code did not seem to be overcome following the activities. In fact, students of both groups tended to disagree with the existence of different start codons (Q7.2) and were shown to be unaware of the existence of a bacterial genetic code (Q7.3), in both pre- and post-test (Figure 6).

These two questions were conceived having in mind that in high school, it is commonly taught that there is a unique start codon, a misconception that is reinforced in most textbooks. During the practical activities, students from the experimental group explored different start codons and worked with a specific bacteria-dedicated genetic code when using the tool NCBI ORFfinder, which was expected to make them aware of the specifications of the genetic code. However, surprisingly, the acquisition of this knowledge was not confirmed, which can be explained by reported evidence that even after being taught and accurately updated on a given scientific content for which misconceptions are observed, many students do not reconstruct their thinking (Mills Shaw et al., 2008). In this study, the practical component designed to address this particular misconception was also not effective. In fact, the use of misleading terms, simplified explanations that induce erroneous interpretations, adapted language, and everyday examples to explain the biological phenomena is often the origin of students’ misconceptions, which can be tenacious and quite difficult to be overcome, ending up being perpetuated all through their high school education (Cho et al., 1985; Soyibo, 1995; Tekkaya, 2003; Mills Shaw et al., 2008). These data call for further attention and suggest that exercises specifically dedicated to exploring different start codons and distinct genetic codes according to the taxa of interest are needed to successfully overcome these deep-rooted misconceptions.

Other knowledge dimensions analyzed in this study include the concepts of genomics (Q4) and comparative genomics (Q5) aimed to acknowledge the importance of genomics in nowadays science and how it is impacting common societal sectors, such as human health and biotechnology. The results recorded for these two questions (Q4 and Q5) revealed a noticeable lack of knowledge about these concepts as previously described (Kirkpatrick et al., 2002; Mills Shaw et al., 2008; Baumler et al., 2012; Chen and Kim, 2014), which bares implications when trying to use bioinformatics tools.

In the post-test, 54.7% of the students in the experimental group provided a correct definition of genomics, i.e., “The field of science that studies genomes,” trend that was not registered in the control group in which only 29.1% of the students were able to define this concept correctly. Zooming in the answers to identify the reported misuse of gene and genomics in an interchangeable way evidences a significant difference between the control and the experimental groups. In the pre-test, 1.5% of the control group students mentioned that genomics is a field of science that studies genes and/or genomes, a frequency that increased in the post-test (5.1%). In turn, in the experimental group, the trend was opposite, with the frequency of these notions decreasing from the pre- to the post-test (8.2 vs. 0.5%). These differences suggest an improvement of the quality of the answers of the students who carried out the bioinformatics exercises, i.e., the experimental group, apparently denoting that the expository teaching failed to clearly teach the difference between genomics and genetics. This may have resulted in the lack of accuracy witnessed in students’ replies to question Q4, in what relates to the reference genome instead of gene. It is important to mention that in the particular case of the Portuguese science curriculum and in the NGSS, genomics is not at all mentioned; the topic addressed when referring to gene- and genome-related issues is genetics. In this regard, before the intervention, only a few students mentioned that they had heard about comparative genomics (Q5; Figure 4), an important concept that currently is not addressed in science classes (Martins and Tavares, 2018).

When students were asked to define comparative genomics in the post-test (Q5.1), the majority was able to do so correctly (79.5% in the experimental group, 75.3% in the control group). They associated the field with “genomic characteristics/genomes/genes/DNA sequences/homologous between different organisms,” which suggests that the expository teaching on comparative genomics was efficient in fostering an accurate understanding about comparative genomics in students in both groups. As comparative genomics was a notion new to students, it was not conditioned by their previous perceptions, contrary to what happened with the concepts of genetics and genomics. Despite this general trend, question 5.1 was also aimed to depict more misconceptions that could be associated with the definition of comparative genomics. In the pre-test, 3.6% of the students in the experimental group mentioned that comparative genomics could be defined as comparisons between genes and phenotype, claiming that comparative genomics is the comparison between genetic sequences. The percentages of students with these misconceptions in the experimental and control groups lowered significantly in the post-test (2.4 and 1.4%, respectively). At this stage, i.e., in the post-test, a new notion was identified, with the experimental group students associating comparative genomics with the “Comparison of genomes of two or more species aiming to investigate phylogenetic relations” (6.7%). Having these outcomes in mind, it can be noted that the quality of answers of students in the experimental group improved after the intervention. It is worth mentioning that in the post-test, 5.2% of the control group students also recognized that comparative genomics can be associated with phylogenetic studies, which can be justified by the expositive teaching.

### Attitudes and Interest

Together with the characterization of the students’ knowledge regarding bioinformatics, gene regulation, and genomics, as described in the previous section, a depiction of their attitudes and interest toward bioinformatics was also carried out. As previously mentioned, in the context of this study, interest was interpreted according to Eccles’ expectancy–value model (Eccles, 2005), which foresees motivation as a result of the combination of expectancy and value. The value given by students to a specific task is extremely important because they are more likely to pursue an activity if they acknowledge its worth. The model further differentiates task value into four components: attainment value (importance of doing it correctly), intrinsic value (personal enjoyment), utility value (perceived usefulness for future goals), and cost (competition with other goals; Eccles and Wigfield, 2002; Eccles, 2005; Leaper, 2011).

From the start, students were shown to be aware about the importance of bioinformatics to identify genes (Q3.1). Nevertheless, the classroom discussion that followed the expository teaching session about the need of bioinformatics tools to efficiently mine the huge genomics datasets contributed to reinforce this belief as demonstrated by the statistically significant difference between pre- and post-test results (Figure 6; Table 1).

**Table 1:** Pre- and post-test comparison of students’ knowledge, interest, and attitudes toward bioinformatics.

			Control group				Experimental group			
			*t*	*df*	*p*	*|d|*	*t*	*df*	*p*	*|d|*
Interest	How important do you think bioinformatics is to…(Q3)	…identify genes. (Q3.1)	−6.27	89	<0.01^*^	0.77	−5.94	274	<0.01^*^	0.48
		…store genomic data. (Q3.2)	−1.59	90	0.12	0.21	−2.66	271	0.01^*^	0.21
		…study the evolutionary relations between organisms. (Q3.3)	−1.87	78	0.07	0.22	−3.62	230	<0.01^*^	0.30
	Rate the importance of the following practices (Q9)	Practical work using digital tools in the classroom. (Q9.1)	−1.78	94	0.08	0.18	−1.32	281	0.19	0.08
		Study of genomes and gene regulation in bacteria. (Q9.2)	−1.65	72	0.10	0.17	0.07	209	0.94	0.00
		Study of phylogeny/evolution of bacteria. (Q9.3)	0.48	62	0.63	0.15	−1.64	221	0.10	0.09
		Using bioinformatics tools in the class. (Q9.4)	0.00	41	1.00	0.03	1.33	181	0.18	0.12
Knowledge	Rate your agreement with the following statements (Q7)	Different taxonomic groups of bacteria have genes in common. (Q7.1)	−4.12	66	<0.01^*^	0.40	−6.08	182	<0.01^*^	0.47
		There are different initiation codons. (Q7.2)	0.99	92	0.33	0.11	−1.78	279	0.08	0.13
		There is a specific genetic code for bacteria. (Q7.3)	0.32	65	0.75	0.02	−1.61	191	0.11	0.17
		All the bacterial genes are known. (Q7.4)	−0.22	66	0.83	0.05	0.00	207	1.00	0.00
Attitudes	How often do you use the computer/technological devices…(Q8)	…for autonomous study outside the classroom. (Q8.1)	0.20	94	0.84	0.02	1.72	290	0.09	0.11
		…in the classroom to study. (Q8.2)	−1.86	94	0.07	0.16	−5.03	290	<0.01^*^	0.30
		…to access bioinformatics tools outside the classroom. (Q8.3)	0.28	94	0.78	0.04	−2.82	285	0.01^*^	0.20
		…to access bioinformatics tools in the classroom. (Q8.4)	0.00	94	1.00	0.00	−11.89	286	<0.01^*^	0.85

*t*, paired samples t-test for a 95% confidence interval (*p*); *df*, degrees of freedom; *d*, Cohen’s *d* measure of effect size.

* Indicates significant differences between pre- and post-test to each group.

Regarding the role of bioinformatics to store genomic data (Q3.2) and to study evolution (Q3.3), a statistically significant difference was observed from pre- to post-test in the experimental group (Figure 6; Table 1), but not in the control group. As the bioinformatics laboratories entailed the recruitment of bioinformatics resources particularly suited to access large datasets and address evolutionary inferences through synteny maps, these results highlight the direct impact of the intervention, which sustains identical results detailed in other studies (Luscombe et al., 2001; Kremer et al., 2005).

When asked to rate the importance of studying gene regulation (Q9.2) and evolution in bacteria (Q9.3), students in both groups agreed on its importance in both assessment moments (Figure 6). In what concerns the study of gene regulation (Q9.2.1), in the control group, its perceived importance was mainly connected with its usefulness from an instrumental point of view (60.3%; utility value), as suggested by expressions that linked its importance with the goals, such as “To get in touch with the world around us” or “To improve human life quality.” Interestingly, in the experimental group, adding to the utilitarian value (42.7%), a more knowledge-related intrinsic worth (intrinsic value) was also well represented (42.7%), as shown by statements, such as “When we study bacteria it is interesting to have the chance to better understand this group and to get information about their metabolism in different environments.” These results indicate that the scientific topic chosen for these activities is of interest to the students, and that the bioinformatics exercises carried out by the experimental group contributed to a more focused appraisal of the relevance of genomics and gene regulation. An identical trend was observed concerning the interest of evolutionary studies in bacteria (Q9.3.1), with 59.0% of the students in the experimental group and 50.9% of the students in the control group mentioning notions that reflect their motivation to explore the scientific topic, which emphasizes the importance of adding comparative genomic tools to the activities proposed.

As expected, students considered the practical work using digital tools important, engaging and motivating, raising their intrinsic interest (Q9.1, Q9.1.1; Figure 6). Concerning students’ interest on the use of bioinformatics tools in the classroom, even before the *in silico* laboratories, they had already shown to be motivated in this regard (Q9.4; Figure 6). Despite the lack of statistically significant differences (Table 1), in the post-test, the students from both groups agreed that the integration of bioinformatics laboratories in the classroom (Q9.4) can have a beneficial impact to increase their intrinsic interest. This suggests their curiosity and awareness about the potential of using these tools in the classroom, regardless of whether they carried out (experimental group) or not (control group) the bioinformatics exercises.

Interesting remarks on the participants’ engagement and interaction can be made based on the observations carried out during the implementation of the activities. For instance, the students were very surprised when they realized the incredible amount of open-access biological data, as translated by questions of amazement, such as “Can I access these bioinformatics resources for free at home?” and “Nice! Everyone can do it?.” Having in mind we are now living in the post-genomic era, these reflections are crucial for students to get acquainted with genomics data sharing and to become aware of the social benefits and ethical implications of open access data (Foster and Sharp, 2007; Oliver et al., 2012).

Another aspect that students stated as being truly interesting pertained to the fact that they were sharing the exact same platforms used by professional researchers. These findings meet the reported importance of exposing science students to real-world phenomena and data, since this kind of activities can increase their interest and better prepare them for engaging in careers in science (Gelbart and Yarden, 2006; Flanagan, 2013). Furthermore, the observations showed that after completing the activities, students looked forward to exploring other tools in the platforms suggested, making comments, such as “What is the size of the genome of a spider?,” “Are virus – such as HIV, genomes also available at this database?,” or “Let us search for the gene coding for insulin.” While this enhanced enthusiasm and curiosity have been reported for university science students (Chapman et al., 2006; Madlung, 2018), it has been poorly described in pre-university levels of education, which makes this finding even more interesting.

Confirming the participants’ interest in learning science with bioinformatics tools is the fact that only a low percentage of students (13.5% in the experimental group, 9.3% in the control group) associated the integration of bioinformatics in the class (Q9.4.1) with a cost, according to Eccles’ framework (Eccles, 2005). These students mentioned that incorporating bioinformatics in the classroom “is not that important once there are similar ways of obtaining the same results” or that “According to the Portuguese curricula for science in high school there is no need of using such complex tools” and also “This kind of activities can make classes more confusing since students are not used to working with these applications.” These comments seem to reveal a lack of sympathy for innovative learning challenges.

As it is well-known, nowadays, youths are particularly at ease with digital resources (Rosen et al., 2010; Quinn and Oldmeadow, 2013), and indeed, students from both the experimental and the control groups admitted that they often take advantage of the technologies at their disposal in their autonomous study outside the classroom (Q8.1; Figure 6). Despite this reality, students from both groups stated that they do not use computers or other technological devices in the classroom (Q8.2, Figure 6). The statistically significant pre- to post-test increase observed in the answers to this question among the experimental group students is likely due to the unique opportunity created by this study for them to join bioinformatics laboratories (Table 1). Recent studies reported that although schools apparently have the necessary conditions to successfully integrate Information and Communications Technology (ICT) in the classroom, there are still barriers, such as teachers’ pedagogical beliefs, which prevent the use of computers in classroom settings (Marcinkiewicz, 1993; Ertmer, 2005; Sang et al., 2010). Interestingly, some informal comments made by the students revealed that their teachers often feel discouraged to use technology in the classroom because they do not feel comfortable with it, which meets the constraints mentioned by the teacher, the majority of whom acknowledged their anxiety regarding the use of technology in this setting (Machluf and Yarden, 2013; Martins et al., 2018c, 2020).

Even though students of both groups also revealed (Q8.3) that they usually do not access bioinformatics tools outside the classroom, there is a significant pre- to post-test difference for the experimental group, which may suggest that these students decided to take advantage of the bioinformatics resources explored after the activities (Figure 6; Table 1). Regarding the specific use of bioinformatics tools in the classroom (Q8.4), while in the pre-test, students from both groups answered negatively to this question, as expected, in the post-test, the students from the experimental group reported that they used bioinformatics in their classes (Figure 6; Table 1).

Having in mind that the students who took part in this study belong to a highly technological society, one can anticipate that their performance in manipulating computer-based tools was efficient (Rosen et al., 2010; Quinn and Oldmeadow, 2013). Indeed, and regardless that most of the students had never experienced working with bioinformatics tools before, during the implementation of the bioinformatics laboratories, no major difficulties to follow the guidelines and discussing the issues raised were reported to the teacher. The observations showed that students were completely able to manage the platforms and did not feel the need to use printed out guidelines. Instead, they looked for solutions and alternatives together with their classmates and took advantage of the technological resources available, namely, smartphones. In spite of the expectable side talk, the participants’ behavior and their questions and comments suggest their engagement in every task that they were asked to perform.

## Conclusion

The findings obtained in this study demonstrate an improvement in students’ knowledge of concepts, such as gene, protein synthesis, nucleic acid (DNA, RNA), start and stop codons, genome, evolutionary relations, and genomic or comparative genomics, following their participation in bioinformatics-based activities “Mining the Genome: Using Bioinformatics Tools in the Classroom to Support Students Discovery of Genes” (Martins et al., 2018a). By the end of the activities, students were also shown to be more aware of the applications and potential of bioinformatics.

This study also raises several questions that are worth pursuing in future research, namely, related with misconceptions that were addressed in this intervention. In addition, future focus on other school levels (namely, middle school) and other curricular topics might be relevant to cross-examine and more widely and consistently depict the impact of bioinformatics-based activities in the classroom. Likely pertinent will be to assess the influence of the “teacher” in students’ performance through a nested effect analysis.

Beyond the evidence of the educational benefits of incorporating practical activities in science education programs, overall, this study represents a contribution to introduce a top-notch research area – bioinformatics – in school and to inform stakeholders about its potential from not only educational but also scientific and other social points of view.

## Data Availability Statement

The raw data supporting the conclusions of this article will be made available by the authors, without undue reservation.

## Ethics Statement

The project was institutionally approved by each school’s Directive Board. Written informed consent from the participants’ legal guardian/next of kin was not required to participate in this study in accordance with the national legislation and the institutional requirements.

## Author Contributions

AM, MJF, and FT designed the research plan. AM followed the implementation of the instruments in the classroom and collected, organized, and analyzed the data. MJF, ML, LL, and FT participated in the data analysis and interpretation. All authors contributed to the writing of this article and approved the submitted version.

### Conflict of Interest

The authors declare that the research was conducted in the absence of any commercial or financial relationships that could be construed as a potential conflict of interest.

## Footnotes

^1^https://drive.google.com/file/u/1/d/1WrtjzLHzKI7nLALtVnmqy6WhPnkUsQSR/view

^2^https://bioinformaticaaula.wixsite.com/bioinformatica-pt

^3^https://drive.google.com/file/u/1/d/1WrtjzLHzKI7nLALtVnmqy6WhPnkUsQSR/view
