# Supplementary material for: Bioinformatics-Based Activities in High School: Fostering Students’ Literacy, Interest, and Attitudes on Gene Regulation, Genomics, and Evolution
Source: Front Microbiol. 2020 Oct 14;11:578099. doi: 10.3389/fmicb.2020.578099 (PMC7591593; doi:10.3389/fmicb.2020.578099)
Supplement: Supplementary file 1 [file Data_Sheet_1.pdf]

**Question**	**Q1.1:** Describe what is bioinformatics for you.	**Q2.1:** Indicate the main procedures that would follow to identify the gene(s) present in that sequence.	**Q4:** What is genomics for you?	**Q5.1:** Define comparative genomics.
**Intervention Aim**	Keep students in touch with the emerging field of bioinformatics.	Highlight the procedures needed to identify a gene in a genomic sequence using bioinformatics tools.	Teach the definition of genomics.	Teach the definition of comparative genomics.
**Question Aim**	Diagnose the students' conceptions about bioinformatics.	Appraise the procedures students describe to identify a gene in a genomic sequence.	Diagnose the students' conceptions about genomics.	Diagnose the students' conceptions about comparative genomics.
**Answers' categorization system**	⇒ Etymology<br>⇒ Applications:<br>→ Data Analysis<br>→ Data Storage<br>→ Comparative Genomics<br>⇒ Other conceptions:<br>→ Technology<br>→ Learning tool<br>→ Interdisciplinary	⇒ Expected Answer *(bioinformatics tools)*<br>→ Get a DNA sequence from a database<br>→ Looking for Open Reading Frames using ORFfinder tool<br>→ Running a BLAST of different ORF's.<br>⇒ Other procedures (examples)<br>→ Performing an electrophoreses to determine the genes<br>→ Looking at the gel bands and comparing with a reference gene<br>→ Restriction enzymes can be needed	⇒ Expected Answer<br>→ Science that studies the genomes.<br>⇒ Misconceptions<br>→ Bioinformatics is synonym of genomics.<br>→ Gene and genome are the same/students use the terms indifferently.<br>→ Genetics (study of genes) is the same that genomics (study of genes).<br>⇒ Other conceptions	⇒ Expected Answer<br>→ Genomic characteristics/genomes/ genes/DNA sequences/comparison of homologous.<br>⇒ Misconceptions<br>→ Comparison of genes and their phenotypic product.<br>→ Comparison between specific genetic sequences.<br>⇒ Other conceptions

Supplementary Figure 1. Open-ended questions and answers' categorization system used to perform content analysis regarding knowledge dimension.
