# Supplementary material for: Bioinformatics-Based Activities in High School: Fostering Students’ Literacy, Interest, and Attitudes on Gene Regulation, Genomics, and Evolution
Source: Front Microbiol. 2020 Oct 14;11:578099. doi: 10.3389/fmicb.2020.578099 (PMC7591593; doi:10.3389/fmicb.2020.578099)
Supplement: Supplementary file 2 [file Data_Sheet_2.pdf]

Supplementary Table 1. Comparison between pre- and post-test students' knowledge towards bioinformatics, gene regulation and genomics.

		Control Group (Pre-test vs. Post-test)			Experimental Group (Pre-test vs. Post-test)		
		n	*p*	*Phi*	n	*p*	*Phi*
**Knowledge**	Q1: Have you heard about bioinformatics?	94	-	-	292	<0.01*	0.08
	Q2: Imagine the following situation: "As a researcher, you sequence a genomic fragment. Do you have any idea how you would proceed to identify the gene (s) present?"	95	<0.01*	0.21	281	<0.01*	0.20
	Q5: Have you heard about comparative genomics?	94	<0.01*	0.11	289	<0.01*	0.08
	Q6.1: Databases are free access resources.	25	0.04*	0.32	105	<0.01*	0.19
	Q6.2: All citizens have access to the main genomic databases.	52	0.02*	0.28	165	<0.01*	0.14
	Q6.3: All bioinformatics tools require programming skills.	80	<0.01*	0.21	223	<0.01*	0.08
	Q6.4: Bioinformatics tools are essential to molecular biology studies.	62	0.22	0.25	199	0.52	0.21

n – number of participants; McNemar test for a 95% confidence interval; Phi – Phi coefficient measure of effect size. (*) indicates significant differences between pre- and post-test to each group.
